# MORTALITY OF URGENCY VERSUS ELECTIVE VIDEOLAPAROSCOPIC CHOLECYSTECTOMY FOR ACUTE CHOLECYSTITIS

**DOI:** 10.1590/0102-6720201700010013

**Published:** 2017

**Authors:** Saulo José Oliveira FELÍCIO, Ediriomar Peixoto MATOS, Antonio Maurício CERQUEIRA, Kurt Wolfgang Schindler Freire de FARIAS, Ramon de Assis SILVA, Mateus de Oliveira TORRES

**Affiliations:** Bahiana Medical School and Public Health, Salvador, BA, Brazil

**Keywords:** Laparoscopic cholecystectomy, Cholelithiasis, Cholecystitis

## Abstract

**Background:**

Surgical approach is still controversial in patients with acute cholecystitis: to treat clinically the inflammatory process and operate electively later or to operate immediately on an emergency basis?

**Aim:**

To test the hypothesis that urgent laparoscopic cholecystectomy in acute cholecystitis has a higher mortality than elective laparoscopic cholecystectomy.

**Methods:**

From the data available in Datasus, mortality was compared between patients undergoing elective laparoscopic cholecystectomy for cholelithiasis and in urgency. Calculations were made of the relative reduction in risk of death, absolute reduction of risk of death and number needed to treat.

**Results:**

From 2009 to 2014 in Brazil, there were 250.439 laparoscopic cholecystectomy and 74.6% were electives. Mortality in the emergency group was 4.8 times higher compared to the elective group (0.0023% vs. 0.00048%). Despite the relative reduction in risk of death (RRR) was 83%, in the calculation of absolute risk was found 0.0018 and number needed to treat of 55,555.

**Conclusions:**

Despite the relative risk reduction for mortality was high comparing elective vs. urgent basis, the absolute risk reduction was minimal, since this outcome is very low in both groups, suggesting that mortality should not have much influence on surgical decision.

## RESUMO

**Racional:**

Continua controversa a conduta nos pacientes com colecistite aguda: compensar o processo inflamatório e operar eletivamente ou operar imediatamente em caráter de urgência?

**Objetivo:**

Testar a hipótese de que a colecistectomia videolaparoscópica de urgência por colecistite aguda apresenta maior mortalidade que a colecistectomia videolaparoscópica eletiva

**Métodos:**

A partir dos dados disponíveis no Datasus, foi comparada a mortalidade entre os pacientes submetidos à colecistectomia videolaparoscópica eletiva por colelitíase e a de urgência. Foram realizados cálculos da *redução relativa de risco de morte, redução absoluta do risco de morte e* n*úmero necessário para tratar* .

**Resultados:**

De 2009 a 2014 no Brasil, foram realizadas 250.439 colecistectomias videolaparoscópicas sendo 74,6% eletivas. A mortalidade no grupo de emergência foi 4,8 vezes mais elevada em comparação com o grupo eletivo (0,0023% vs. 0,00048%). Apesar da redução relativa do risco de morte (RRR) ser de 83%, no cálculo do risco absoluto encontrou-se 0,0018 e número necessário para tratar de 55.555.

**Conclusões:**

Apesar da redução relativa de risco para mortalidade ser alta comparando o caráter eletivo vs. urgência, a redução de risco absoluto é mínima, já que esse desfecho é muito baixo nos dois grupos, sugerindo que a mortalidade não deve ter muita influência na tomada de decisão cirúrgica.

## INTRODUCTION

In Brazil, cholelithiasis represents a surgical condition with 9,4% of global prevalence, with increased incidence in elderly and upwardly by increasing obesity^13^.

In 1992 the National Institute of Health of the USA defined cholecystectomy by laparoscopy (VLC) as a choice in the treatment of biliar lithiasis. On the opposite way of this recommendation, in 2013 in Brazilian's Unique Public Health System (SUS) there was predominance of laparotomy cholecystectomy in comparison to VLC in country's regions^4^ ^,^ ^5^ ^,^ ^14^. 

VLC is more aesthetic, reduces postoperative pain, wound infection, length of hospital stay, morbidity, mortality (0.1%), and can be done on an outpatient basis. The return to work activities usually occurs after 7-10 days. Generally has a low incidence of complications, but there is the possibility of conversion to laparotomy with increased morbidity and mortality as a function of the severity of inflammation^2, 8,17,18,19,22^. 

A study conducted in a general hospital in the United Kingdon^22^ compared urgent with elective videolaparoscopic cholecystectomy. Analysig 2117 patients with gallstones, there was predominance in elective in 80.6%; the meantime, in the elective procedure was around 59 min vs. 69 min in the emergency. Regarding complications, the most frequent were 3.4% of the bile leaks in the urgency versus 1.0% in elective; 4.9% of cardiorespiratory complications in the urgency vs. 1.5% in elective. The risk factors for conversion to laparotomy, in elective was 5.9% while in urgency was 6.3%. In 2013 a Cochrane review showed that the increased incidence of morbidity should not prevent the use of early VLC for acute gallbladder disease because the procedure still would be safer than the operation done later^7^. Elective or emergency, the fact is that there is small amount of publications comparing these two periods of service; moreover, they were not found reports in the literature about VLC in relation to the Unified Health System (SUS) of Brazil as a whole.

The aim of this study was to test the hypothesis that emergency laparoscopic cholecystectomy presents higher mortality than elective VLC.

## METHODS

A descriptive study of hospital mortality compared this indicator to elective and urgent VLC. This information was obtained from the database of the Ministry of Health, Datasus (SUS Department of Informatics). Data from AIH (Hospitalization Authorization) paid corresponding to admissions made in January 2009 to December 2014, non-probability sampling of the associated network and own network of SUS were used.

Approval by the Research Ethics Committee was not required because the data collected is in the public domain, free and unrestricted access without individual identification of patients.

The inclusion criteria were: patients undergoing VLC diagnosed by International Classification of Diseases Chapter 10 (ICD10) of gallbladder calculi with acute cholecystitis K80.0; calculi gallbladder without cholecystitis (cholelithiasis symptomatic K80.2); and cholelithiasis asymptomatic K80.8. Patients submitted to VLC with diagnostics for the acalculous conditions K81, K82; with cholangitis associated K80.3, K80.4; with chronic cholecystitis K80.1 and disease in other locations in the bile duct K80.5, K83, were excluded.

The information obtained and arranged in tables by Datasus own tabulator were the following variables: gender, care character, days of hospitalization in general or in the intensive care unit, nine types of age, number of deaths in general or ICU. These data were divided into two groups: patients undergoing emergency VLC and elective VLC. In emergency VLC group were only included patients with a diagnosis of acute cholecystitis lithiasic; other diagnoses were excluded. In elective VLC were only included patients with symptomatic cholelithiasis, asymptomatic and the others were excluded.

The age range was organized as follows: <1 year (y) 1-4y, 5-14y, 15-24y, 25-34y, 35-44y, 45-54y, 55-64y and ≥65y. The length of stay was in days, deaths and use of intensive care. The emergency data were analyzed after breakdown by age group, keeping in days, deaths and use of intensive care. After the breakdown by age group, the data were used to calculate the mortality and mean hospital stay. Equally it proceeded with the data of patients undergoing elective VLC. From there, the data of elective and emergency group, was distributed by age group and were compared by gender alone and each other. In the comparison between elective and emergency were performed relative reduction calculations of risk of death, absolute risk reduction and number needed to treat using standard formulas.

## RESULTS

From 2009 to 2014 it was performed 250.439 VLC being 74.6% elective cholelithiasis and 25.4% of urgency. For the study, after they apply the exclusion criteria, 98,716 patients were studied, 12,197 (12.4%) emergency being 86,519 (87.6%) elective.

In the elective group (Table 1) in a population of 86,519 patients 82.8% (71,652) were women, with a predominance of the age group 45-54 years (22.4%). The mortality was 42 patients, representing 0.00048% of the total, nine were men (0.0006) and 33 women (0.0004%). It was found that 760 patients (0.0087%) required hospitalization in the ICU, with a predominance of women (69%), but in relative terms, men go to the ICU twice more than women (0.015% vs. 0.007%). The average number of days of hospitalization in ICU was 7.04 days.

**TABLE 1:** Patient outcomes: elective surgery

F.E.	n		MP		O		UTI		MP. UTI		O.UTI	
	F	M	M	F	M	F	M	F	M	F	M	F
<1a	18	61	3,1	3,1	0	1	0	0	0	0	0	0
1-4a	61	46	2,3	2,3	0	0	1	0	3	0	0	0
5-14a	428	758	2,8	2,6	0	0	6	5	4	6,8	0	0
15-24a	668	5277	2,3	2,1	0	2	4	15	9,8	9,3	0	2
25-34a	1578	12759	2	1,9	0	0	4	26	22,2	7	0	0
35-44a	2707	14617	2,1	2	1	3	12	36	13,7	6,7	0	0
45-54a	3365	16024	2,1	2	3	3	26	76	6,9	5,5	1	1
55-64a	3301	13327	2,2	2,1	2	3	56	106	5,9	8	2	2
≥65a	2741	8783	2,8	2,3	3	21	121	266	6,2	5,4	0	15
Total	14867	71652	2,3	2	9	33	230	530	5,8	6,2	3	20

F.E=age range; n=total of patients; MP= average stay (days); O=deaths; UTI=total of patients that needed UCI care; MP.UTI=average permanence in intensive care by patients (days); O.UTI= deaths on UCI; M=male; F=female; a=years.

In the group of patients operated on an emergency basis (Table 2), the population was 12,197 people, in which 76.4% (9,326) were women, with a predominance of the age group between 35-44 years (20.5%). The mortality rate was found in 29 patients (0.0023%), eight men, corresponding to 0.0027% of men and 21 women, corresponding to 0.0022% of women in this group. Hospitalization in ICU beds were verified in 237 patients (0.019%), predominantly women in absolute values (59%). The average hospital stay was similar to elective VLC group: 7.3 days.

**TABLE 2:** Patient outcomes: urgent surgery

F.E.	n		MP		O		UTI		MP. UTI		O. UTI	
	M	F	M	F	M	F	M	F	M	F	M	F
<1a	3	7	2,7	3,7	0	0						
1-4a	6	7	2,7	2,6	0	0		1		3		0
5-14a	55	85	3,6	4,2	0	1	1	3	4	7,7	0	1
15-24a	150	925	4,8	4,8	0	0	3	5	9,3	8	0	0
25-34a	338	1917	4,4	4,1	0	3	1	11	20	7,7	0	1
35-44a	572	1937	4,8	3,9	0	1	8	10	12,1	7,2	0	1
45-54a	629	1855	4,8	4,2	0	3	5	17	8,4	10,2	0	3
55-64a	574	1466	5,4	4,8	0	5	20	24	7,7	10	0	2
≥65a	544	1127	7,2	5,6	8	8	58	70	11,5	10	5	3
Total	2871	9326	5,3	4,4	8	21	96	141	10,5	9,5	5	11

F.E=age range; n=total of patients; MP= average stay (days); O= deaths; UTI= total of patients that needed UCI care; MP.UTI=average permanence in intensive care by patients (days); O.UTI=deaths on UCI; M=male; F=female; a=years.

Mortality in emergency VLC is about five times higher than elective, with a relative risk is 0.17, and relative risk reduction of 83% kill, or reduce the risk of mortality by 83% when the VLC is elective and non-emergency. However, the absolute risk is equal to 0.0018, which promotes the number needed to treat equal to 55,555 (NNT = 55,555), representing minimum benefit when comparing the two intervention scenarios. In relation to the ICU stay, in general, the emergency surgery interned twice more than elective, equal numbers of men and women.

## DISCUSSION

Predominated elective treatment (87.6%) higher value than in studies conducted in the UK, in which were described percentage from 71.1%^9^ to 80.6%^22^ to elective procedures. The females compared to males presented 4.9:1 for elective and in urgency 3.2:1, in the present study. In Hinchingbrooke Hospital, United Kingdom, the ratio found to elective was 2.8:1 vs. emergency 3.1:1^9^. Therefore, there is a greater amount of women and elective surgeries in this study compared to literature; this may be related to the fact that in Brazil men have greater resistance to go to medical appointments, so more difficult to be diagnosed.

In the analysis of patients undergoing surgery for age, there was progressive increase in the incidence of operations only up to 54 years; from 55 years there was slight decrease in numbers of emergency and elective. In the literature it is observed that the gallstones is rare in children, begins to be identified in adolescence; shows marked increase in incidence between 35-55 years and increases gradually after 55 years of age^4^. Emeline Caldana et al found in their study a very low incidence of cholecystitis and cholelithiasis in patients between 0-4 years, with zero mortality^16^. The number of operations should increase with age, as gallstones increases with it, but this did not happen; perhaps, the risk of cholelithiasis and the risk of being submitted to VLC differ because there is less symptoms in older ages.

Patients over 55 have lower chances to go through emergency procedure in relation to the younger. K. Saeb-Parsy Et al showed that elective patients were also significantly older than the emergency, mean age 53.1±14.5 y vs. 49.2±16.1 y^9^; these findings are consistent with those found in this study. Probably symptomatic patients are operated before these ages.

In the age groups analyzed, elective surgery was most commonly used in relation to the urgency and, in general, keep the lowest mortality, namely the elective/emergency as 0.00048% and 0.0023%, respectively. K. Saeb-Parsy Et al analyzed all emergency and elective admissions related to gallstones in the diagnosis of biliary colic, cholecystitis or biliary pancreatitis submitted to emergency VLC during the same admission^9^. The average mortality rate in elective patients was 1.6% and significantly lower than in emergency situations 2.6% (p<0.001)^9^. Perhaps the mortality differed by having inserted in the sample other lithiasic disease, such as acute biliary pancreatitis.

Another parameter the hospital permanency time shows that emergency surgical patient spends almost in all age groups the double time of hospital stay for both genders. There are important risk factors for infectious complications in VLC including: acute cholecystitis, common bile duct stones, emergency procedures^11^. Procedure in character of urgency is a risk factor for complications. 2013 Cochrane Library review suggests that studies with high risk of bias indicate that early VLC during acute cholecystitis seems safe and can shorten the stay in hospitais^10^. In this study, the emergency procedure seems to be more morbid that elective, unlike the cited in the review. But, there is also this study limiting factors for analysis, as far as it was not possible to evaluate preoperative hospital stay, or the presence of comorbidities, to analyze the quality of life after the operation because patients often have multiple hospitalizations for clinical lithiasis compensation before the operation.

When assessing mortality and complications of patients undergoing emergency VLC and compares with elective, it is noted that mortality have a very low absolute value for both groups, increase in time on the operation made in urgency character suggests major complications in the VLC. There are meta-analyzes of randomized clinical studies that say otherwise and suggest that early VLC is safer in selected cases and is not associated with increased complications in relation to the delay of VLC, which goes against the idea that the VLC should expect the acute picture pass on diseases related to calculi biliar^1^ ^,^ ^7^ ^,^ ^10^ ^,^ ^20^ ^,^ ^21^. In the present study the analysis of complications and morbidities resulting procedures was impaired because patients treated medically that were not operated also had complications and mortality. Also mortality of acute cholecystitis when operated emergency can increase the length of stay, if no offer treatment of acute cholecystitis in the first hospitalization for worsening of disease. Thus, the patient will be admitted for clinical treatment and then will be admitted for elective surgery. For a better view, we would need to compare mortality of operation in emergency with mortality of elective surgery combined with the mortality of treated medically and this is not available in Datasus.

The waiting list for elective surgery in Brazil is something to be considered because patients are operated electively according to order established by outpatient care. Those with important symptoms of biliary disease, in this waiting period, show up in emergency and urgent services and can be admitted, readmitted and treated until remission of symptoms. They are discharged and return to their homes, often without being operated, so no definitive solution was done to the underlying problem. The readmission rate of return of symptoms in patients in an elective waiting list for VLC is estimated in the literature between 5-39%^9^.

So in Brazil often they are not operated at the time of emergency symptoms. K. Saeb al-Parsy Et confirm that all patients who present acutely with disease related to gallstones should be considered for emergency VLC whenever possível^5^. In a review of literature on acute cholecystitis of the University Hospital Pedro Ernesto team in Brazil, indicates early operation because there is difficulty of getting beds for elective^12^.

Men, despite being the minority gender in the study, are more operated on an emergency basis in relation to women. Studies suggest that the male gender is a risk predictor in laparoscopy for chronic cholecystitis; it increases the incidence of intraoperative incidents and accidents that is in men 32% and in women 2.9%^14^ ^,^ ^21^. Perhaps being a man is a predictor of risk for acute cholecystitis.

In SUS is noted that the overwhelming majority of procedures performed on an emergency basis, much increase the hospitalization time. When offered the first exacerbation, VLC allows shorter hospitalization and is profitable. However, early VLC is not commonly practiced and patients may be readmitted up to ten times before undergoing VLC^4^ ^,^ ^10^ ^,^ ^20^. It is possible that many SUS patients are hospitalized multiple times by acute exacerbation and cleared medically before definitive treatment, eventually complicate and enhance the morbidity and mortality in emergency.

The percentage of patients undergoing surgical treatment of first admission is difficult to measure in the SUS since it does not have access to stunted patient information. VLC in the first acute presentation of diseases related to gallstones can decrease readmission rates, increases patient satisfaction without compromising security. There may be emergency VLC contribution to reduce the risk of progression of biliary disease with obstructive jaundice, biliary pancreatitis or ascending cholangitis^9^. In about 25,000 acutely patients admitted to hospitals in England with gallbladder disease between April 2003 and March 2005 showed that only 14.7% were submitted to VLC during initial admissions^4^. This difficulty exists in obtaining that information because there is no access to the preoperative hospitalization of patients; so it is not possible to know whether the patient was operated in the first 24 h of disease onset.

The information of patients who were waiting for elective surgery and passed to urgency is unavailable in the study. In 27.0% of VLC, patients were already on the waiting list for elective surgery and about 11.5% in the emergency group were on the waiting list for more than 90 days before emergency VLC^4^. This information is interesting from the monetary and social costs affairs, to the taxpayer and to the health system as a whole. 

Do the emergency surgery in the patient with acute cholecystitis or treat him clinically to operate after as elective for cholelithiasis?

When we analyze the amounts related to the elective and emergency (0.00048% vs. 0.0023%), and compare the two interventions it is clear that mortality in emergency VLC is about five times higher, giving relative reduction of mortality risk in 83%, that is the risk of mortality is reduced by 83% when the VLC is elective and not in emergency. The first impression is that the benefit, in fact, is very expressive. However, in the medical context, to judge the magnitude of a benefit must be used absolute concepts rather than relative ones. Thus, it is clear that the calculated absolute risk is 0.0018. Going beyond the "absolute" concepts, one can calculate the number needed to treat equal to 55,555 (NNT=55,555), showing that indeed, the magnitude of the benefit-related to mortality is very low. This occurs because the mortality in the two groups is quite small

Despite the limitations of the study, a detailed analysis of the results is important generator of hypothesis and important points of reflection. Studies are needed to add as clinical admissions for acute exacerbation of diseases and how much operations in the first exacerbations account for mortality and morbidity in elective and emergency patients.

## CONCLUSIONS

Despite the relative risk reduction for mortality is high, comparing the urgent vs. elective VCL, the absolute risk reduction is minimal, since this outcome is very low in both groups, suggesting that mortality should not have much influence on decision-making in this context.
